# SODS: Soil Health On-Demand Sensors—A Multi Parameter Field Study with Temporal Monitoring

**DOI:** 10.3390/s25113505

**Published:** 2025-06-01

**Authors:** Vikram Narayanan Dhamu, Mohammed A. Eldeeb, Anil C. Somenahally, Sriram Muthukumar, Shalini Prasad

**Affiliations:** 1 EnLiSense LLC, Allen, TX 75013, USA; 2 Department of Bioengineering, University of Texas at Dallas, Richardson, TX 75080, USA; 3 Department of Soil and Crop Sciences, Texas A&M AgriLife Research, Overton, TX 75684, USA

**Keywords:** soil sensors, real-time tracking, soil inorganic and organic carbon, soil health, agriculture field temporal study

## Abstract

Real-time monitoring of soil health parameters is crucial for efficient use of resources, improving agricultural productivity, and sustainability. Traditional soil analysis methods, although accurate, are time-consuming and lack the spatial and temporal resolution necessary for dynamic agricultural environments. Recent advancements in sensor technology offer promising alternatives, enabling real-time, in situ monitoring of key soil health indicators. This study details the deployment and validation of novel Sensor-in-Field probes at the Donald Danforth Plant Science Center Farm in Missouri, U.S., in a winter wheat plot. Three Sensor-in-Field probes were evaluated for their ability to measure nitrate (NO_3_), ammonium (NH_4_), soil organic matter (SOM), carbonaceous soil minerals (CSMs), soil volumetric density (SVD), soil hydration state (SHS), and total soil carbon (TSC) over a 28-day period. The probes’ coefficients of variation were well within acceptable limits (<20%) for all parameters. The measured metrics averaged 0.05% ± 0.001 and 1.92% ± 0.02 for CSMs and SOM, respectively, while TSC was 1.18% ± 0.15. For the nutrients, the measured NO_3_ and NH_4_ values were 4.44 ppm ± 0.37 and 2.78 ppm ± 0.22, respectively. The accuracy of the soil probes was validated at a certified traditional soil analysis laboratory. Three samples were collected at three different time points and analyzed. Bland–Altman analysis showed <± 10% difference between the soil probes and traditional lab analysis for CSMs, SOM, and TSC, while *t*-test analysis reported *p*-values > 0.005 for NO_3_, NH_4_, and SHS/SVD, indicating non-significant differences between the probes and traditional soil analysis methods.

## 1. Introduction

Soil health is the measure of soil’s capacity to sustain plant and animal life by regulating water, cycling nutrients, and filtering pollutants. Healthy soils are characterized by balanced nutrient levels, adequate moisture retention, and a thriving microbial community [1]. Without adequate monitoring [1] of soil health, once-fertile agricultural lands dry up into barren deserts. The number of species at risk of extinction due to desertification has risen to about 40,000 [2]. One solution is sustainable agriculture, which is defined as a system that integrates the production of plants and animals that can eliminate food shortages. Efficient use of nonrenewable resources, complemented by the biological cycle, enhances the quality of life for all living organisms while protecting and enhancing the environment’s natural resources [3,4]. To achieve sustainable agriculture, continuous monitoring of the ecosystem and strategically intervening when needed is essential. Continuous real-time soil analysis, in particular, is severely lacking in terms of simplicity, results turnaround, accuracy, and cost. A common technique is image analysis using drone or satellite images [5,6,7,8]. These methods rely on large processing power to analyze thousands of images. Other methods include optical sensors that have good sensitivity; however, they greatly suffer from bulky spectrometer hardware, site-specific calibration, and a lack of accuracy for detecting nutrients that are not fully observed in the Vis-NIR region [9,10]. On the other hand, miniaturized electrochemical sensors require a microcontroller and battery in a handheld device to measure the soil nutrients [11,12,13,14]. Electrochemical sensors have been widely used in soil science and in designing sensing capabilities for measuring soil parameters [6,11]. These sensors have shown discrete temporal measurements. However, in order to build an electrochemical cell, these systems typically require high moisture content, which requires the preparation of soil slurries by mixing the sampled soil with deionized water; hence, these sensors are not compatible for field-based measurements. One big advantage of electrochemical sensors is the high sensitivity and selectivity offered by these systems. The biggest challenges, however, are the variability and relatively poor signal-to-noise ratio with varying soil types and textures.

Of all the electrochemical methods, electrochemical impedance spectroscopy (EIS) is an interesting approach for visualizing and probing multi-layer micro and macro films in a non-destructive manner [15,16,17]. EIS can be utilized to capture a detailed snapshot of the electrical double layer (EDL) that forms at the interface between the electrode surface and the soil matrix [18]. Information about the soil can be extracted by analyzing the EIS data through circuit fitting as laid out previously by our group [18]. EIS measurements also provide better noise-resilience when compared to other soil sensors in the literature that utilize open circuit potential (OCP) to probe the soil [19]. For sensing plant available nitrogen, ion-selective electrode (ISE) technology was developed using non-actin and tridodecylmethylammonium (TDDA) nitrate as the active elements for quantifying ammonium (NH_4_) [20] and nitrate (NO_3_) [19], respectively. For bulk density (SVD) and moisture (SHS), a room temperature ionic liquid (RTIL) was utilized as a transducer on a screen-printed electrode (SPE) where information on the soil’s moisture content and compactness is extracted through circuit fitting of the EIS data [21,22]. Similarly, for the determination of organic matter as an aggregate measure [23], RTIL was used as a transducer to survey the chemical interactions between the organic moieties and the zwitterionic structure in the RTIL (cation/anion pair). To study a largely understudied pool of soil carbon—the inorganic carbon fraction—an ion recognition/capture layer was developed based on Carbonate Ionophore VII from Sigma Aldrich that captures the inorganic carbon pool (fraction containing carbonates and bicarbonates) present in soil for quantifying carbonaceous soil minerals (CSMs) [24]. In-lab validation of all sensors against the standard reference methods yielded error rates below 20%, which are well below the acceptable variation in soil analysis.

This paper presents the first in-field validation of the soil electrochemical sensors that are capable of in situ soil measurements, allowing for continuous, temporal monitoring. This study was conducted to expand on our individual in-lab validated sensors as a suite for accurate in situ monitoring of soil health. Three Sensor-in-Field probes were evaluated for their ability to measure nitrate (NO_3_), ammonium (NH_4_), soil organic matter (SOM), carbonaceous soil minerals (CSMs), soil volumetric density (SVD), soil hydration state (SHS), and total soil carbon (TSC) over a 28-day period. Soil samples were collected at three time points and analyzed to validate the sensor’s accuracy at a certified soil analysis laboratory.

## 2. Materials and Methods

### 2.1. Sensor Calibration

**Figure 1:**
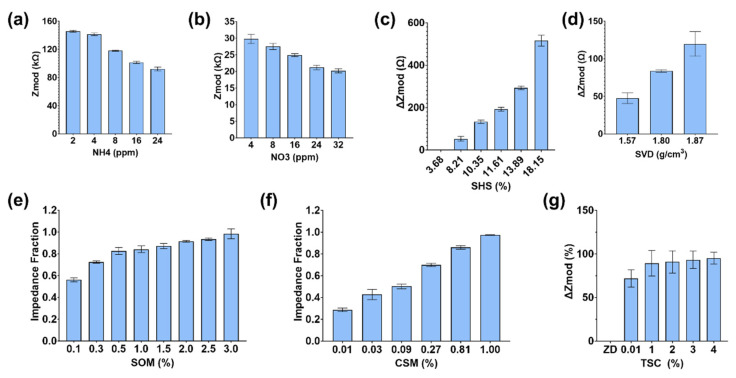
Calibrated dose–response for the 6 sensors used in this study in loamy–clay soil for (**a**) ammonium, (**b**) nitrate, (**c**) soil hydration state, (**d**) soil volumetric density, (**e**) soil organic matter, (**f**) carbonaceous soil minerals, and (**g**) total soil carbon using N = 3 different sensors per parameter. The plots are drawn using the mean ± standard error.

Each sensor was calibrated in loam–clay soil slurry in the lab prior to the start of the field study, with the six calibration curves shown in Figure 1. The soil was air-dried and then sieved using a 2 mm pore-sized sieve to remove pebbles. The soil was spiked with solutions of different concentrations to build the calibrated dose–response model. Sodium nitrate and ammonium chloride were used for the nitrate and ammonium sensors, respectively. A mixture of Humic-Fulvic and Phenolic-Carboxylic was used for the soil organic matter (SOM) sensor, while a cocktail of calcite and bicarbonates was used for the carbonaceous soil minerals (CSMs) sensor. For the soil hydration sensor (SHS), deionized water was added to the air-dried soil using a *v*/*w* ratio to represent the moisture content in the soil. Finally, the soil volumetric density (SVD) sensor was calibrated using three soil cores of loamy clay soil similar to those used in the soil sampling mentioned below.

### 2.2. Soil Probe Setup

**Figure 2:**
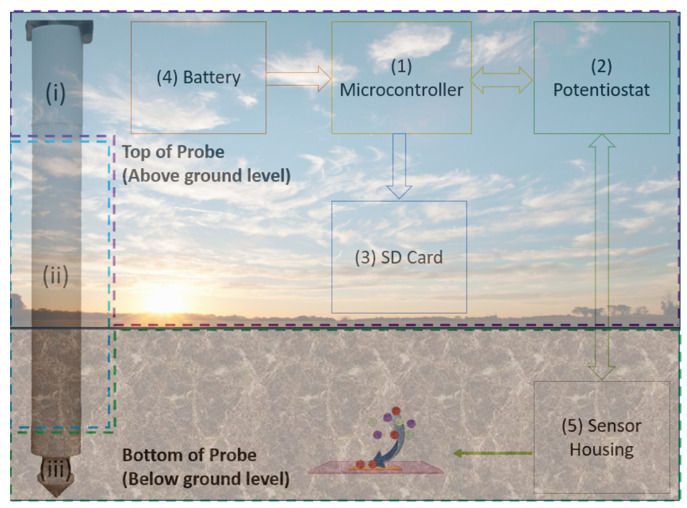
An illustration of the soil probe system showing the 5 key components of the Soil-in-Field Probe. The probe is 46 cm long; compartment (i), where the electronics are housed, is 6 cm long that needs to be above ground for access. Compartment (ii), surrounded by the blue dotted lines, provides flexibility in positioning the sensors from 10 cm down to 40 cm and finally compartment (iii) hosts the sensors. The probes are inserted in a 20 cm deep and 10 cm wide hole, which positions the sensors at a depth of 15 cm to monitor the densest root region. The microcontroller runs an 8 h cycle that serially measures all parameters.

A 3D structure printed using ABS was created to house the electronics and sensors illustrated in Figure 2. The probe has 2 main compartments: the electronics housing and the sensors area. The electronics housing, which sits at the top of the probe, hosts the Arduino MKR zero (component 1, Monza, Italy), EmStat pico module (component 2, Utrecht, Netherlands), an SD card (component 3), and a rechargeable LiPo battery (component 4). At the bottom of the probe lie the sensor slots. The probe is 46 cm long, which provides flexibility in positioning the sensors from 10 cm down to 40 cm. For this study, the sensors are positioned at a depth of 15 cm. The sensors are connected to the electronics via a ribbon cable. The Arduino MKR zero is used as a master to control the EmStat Pico module and store the data on an SD card. Both devices are powered by a single rechargeable Adafruit LiPo 2500 mAh battery (Brooklyn, NY, USA). The Arduino MKR zero was chosen as it comes equipped with an SD card module. Once the battery is connected, the Arduino runs a single measurement cycle, which takes about 10 min to measure all 7 parameters, followed by a hibernation cycle of 8 h. The SD card module is only powered up when saving the data and is then immediately powered down to prolong battery life.

**Figure 3:**
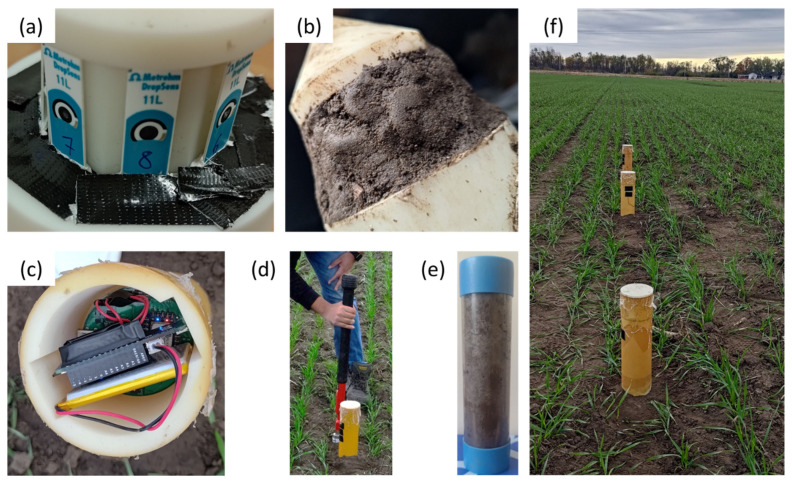
(**a**) A photograph of the sensors after waterproofing the connectors. (**b**) Padding of the sensors with the dug-up soil before insertion ensures contact between the sensors and the surrounding soil. (**c**) The portable data logger and potentiostat are housed at the top of the probe for ease of access to replace batteries on a weekly basis. (**d**) The sampling method used to collect the (**e**) soil cores for analysis at a standard soil-testing laboratory. (**f**) The three Sensor-in-Field probes placed 2 to 4 m apart in a winter wheat plot in St. Louis, MO, USA.

Figure 3 demonstrates the insertion and sampling procedure. First, the sensors are inserted in the designated slots shown in Figure 3a. Then, a waterproof sealing using Teflon tape, followed by gorilla tape, is added on top to ensure perfect insulation of the connectors. A layer of the dug-up soil is gently pressed on the exposed sensors illustrated in Figure 3b to ensure proper contact between the sensor’s surface and the surrounding soil. Afterwards, the probe is inserted into the soil and padded with the remaining dug-up soil. The battery is then connected to the electronics, capped, and sealed with waterproof tape to prevent water from sweeping into the electronics housing. On sampling day, scheduled in Table 1 below, a 2” diameter by 6” long soil core cylinder is manually extracted using a core sample as shown in Figure 3d,e. A total of three samples per probe within close proximity are taken for validation purposes. Figure 3f shows the final probe placement in the field, where they were left undisturbed except when replacing the battery on a weekly basis until the end of the study. The data were then collected on day 28, analyzed, and plotted using GraphPad Prism 10.2.0.

### 2.3. Study Site

Three Sensor-in-Field probes were evaluated for their ability to measure nitrate (NO_3_), ammonium (NH_4_), soil organic matter (SOM), carbonaceous soil minerals (CSMs), soil volumetric density (SVD), soil hydration state (SHS), and total soil carbon (TSC) over a 28-day period. The 28-day study was conducted at the Donald Danforth Plant Science Center Farm, located in St. Charles, MO. According to the USDA-NRCS soil map, the soil series is a Desioux Silt loam with surface soil texture of loam, average pH of 6.5, average SOM of 2.55%, and average SVD of 1.45 g/cm^3^. Three probes were placed two to four meters apart in a winter wheat plot in November, as shown in Figure 3f.

### 2.4. Soil Sampling

Soil samples for laboratory reference analysis were collected within approximately 1 m of individual sensor probes. A sampling probe with a dimension of 8 cm in diameter and 15 cm in length, with the capability to hold plastic liners (a double-cylinder core sampler), was used for soil sampling. A smooth, undisturbed horizontal soil surface was prepared before sampling by removing litter and organic matter. A sample probe with a liner was gently pressed into the soil enough to fill the inner core by applying mild hammering action. The plastic tube liner was removed, ends were capped, and then stored at 4 °C until analysis. A Styrofoam-sealed container with dry ice was used to ship the samples to the soil lab for analysis to reduce fluctuations in the soil’s profile from the sampling time point.

### 2.5. Sample Preparation and Analysis Using the Reference Method

The reference method used for bulk density (BD) estimation is based on the core method [25], which is widely used for estimating BD in agricultural soils [26]. Intact soil cores were first used for BD and soil moisture analysis. Total soil volume and soil mass were estimated to derive BD in g/cm^3^. A subsample of fresh soil was used to determine the soil moisture content and estimate soil dry mass basis. The remaining soil sample was air-dried and stored for further analysis. Sample preparation and analysis protocols for all other analyses followed the details presented in Nelson and Sommers [27]. Thermal oxidation using dry combustion is currently the most reliable and accurate method for determining SOC. Dry combustion at higher temperature (around 1000 °C) ensures oxidation of all carbon to CO_2,_ and subsequently an infrared detector is used for accurately quantifying the amount of CO_2_ [28,29,30]. Samples were analyzed for total soil carbon (TSC) concentration (mg kg^−1^) using a dry combustion instrument equipped with an infrared detector for CO_2_ quantification (a Vario MACRO Elemental Analyzer CN, Elementar Inc., Ronkonkoma, NY, USA). Another set of subsamples was acid-treated (6% H_2_SO_3_) in combustion vessels until effervescence ceased. Samples were then air-dried and analyzed for TSC using the same dry combustion method. This analysis was measured as soil organic carbon (SOC) concentration in mg kg^−1^. Concentrations of carbonaceous soil minerals (CSMs) (also referred to as soil inorganic carbon), which include carbonates and bicarbonates, were obtained by subtracting SOC from TSC. Concurrently, subsamples used for TSC and SOC analysis were oven-dried to estimate soil moisture and correct the final concentrations on a dry mass basis. For quality check and control, a known concentration of carbon chemical standard, one laboratory standard soil, and one reference soil sample were used with an individual set of ten samples. A fixed conversion factor of 1.724 was used to convert SOC to SOM [31].

The reference method used for NO_3_-N and NH_4_-N was based on extracting soil samples using 2.0 M KCl and analyzing N pools using a cadmium (Cd) reduction column analyzer [3]. Approximately 5 g of air-dried soil was added to 20 mL of 2.0 M KCl solution and agitated for 30 min. A reagent blank was included throughout the procedure. The suspension was then filtered through a Whatman No. 42 filter paper. The concentrations of NO_3_-N and NH_4_-N were analyzed using a Cd reduction column analyzer. Statistical analyses of the results received from the sensor and reference methodology were conducted to determine the percentage differences between the two methods.

### 2.6. Data Analysis

Each probe housed six sensors. Measurements were collected once every eight hours daily for a total of 84 data points per parameter per probe. A total of 252 data points per parameter were collected over the 28-day period. The average, standard deviation, and coefficient of variance were calculated after removing outliers. The time series data were plotted by averaging the data across the three probes to show the daily fluctuations in soil health metrics, with the moving average and the lab-analyzed validation results indicated in the figures. All analyses were conducted and figures generated using GraphPad Prism.

### 2.7. GenAI

ChatGPT 3.5 was used to improve the language in a few of the sentences.

## 3. Results and Discussion

**Figure 4:**
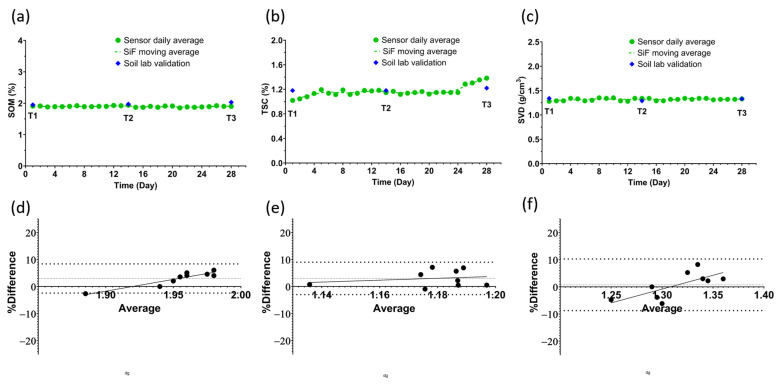
(**a**–**c**) Time series data plotted using the daily mean of 9 measurements: 3 measurements a day across 3 probes. Alongside them are the 6 data points obtained from the analysis of the soil cores by a standard soil testing laboratory. T1, T2, and T3 denote the days when soil cores were sampled and shipped to the soil testing laboratory. The soil health metrics presented are (**a**) soil organic matter, (**b**) total soil carbon, and (**c**) soil volumetric density. (**d**–**f**) Show the Bland–Altman analysis of the lab and sensor data on sampling day, presented as percentage difference on the y-axis and the average on the x-axis. All three parameters have an average difference of less than 4% between the two methods.

**Figure 5:**
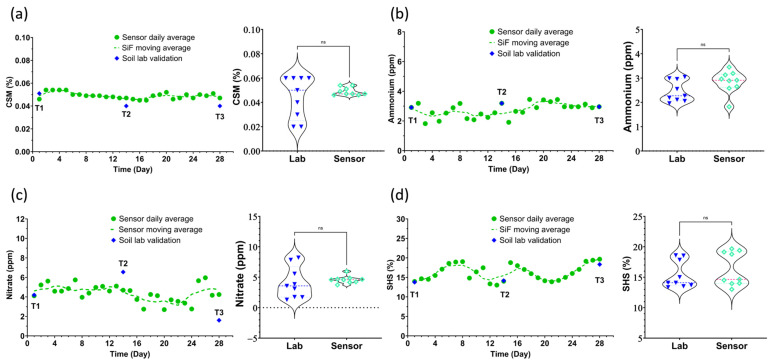
Time series data plotted using the daily mean of 9 measurements: 3 measurements a day across 3 probes. Alongside them are the 6 data points obtained from the analysis of the soil cores by a standard soil testing laboratory. T1, T2, and T3 denote the days when soil cores were sampled and shipped to the soil testing laboratory. The soil health metrics presented are (**a**) carbonaceous soil minerals, (**b**) nitrate, (**c**) ammonium, and (**d**) soil hydration state. The sensor data on sampling day was used to plot violin graphs. *t*-test analysis shows a non-significant difference between the sensors and lab data (ns: *p*-value > 0.005).

Table 1 provides a summary of the soil health metrics recorded by the Sensor-in-Field probes during the 28-day test period, as well as the environmental conditions during the study period. The time series data (Figure 4 and Figure 5) show the fluctuations in soil parameters over days plotted with the daily mean across the three probes. The aim of this study was to determine the premise of in situ capability of soil health measurement and to utilize the context of this study to provide valuable insights into soil health and functionality by means of an active agricultural field example—winter wheat plots with a silt loam soil texture. The validation aspect involved comparing Sensor-in-Field probe readings with reference laboratory methods.

Key takeaways from the field validation study conducted at the Donald Danforth Plant Science Center Farm related to soil nutrient levels. NO_3_ levels displayed low availability values across multiple probes, averaging around 4.40 ppm, and NH_4_ concentrations were consistent, averaging 2.78 ppm. Similarly, soil carbon information was examined, with SOM content at 1.9%, carbonaceous soil minerals (soil inorganic carbon) levels at 0.05%, and overall TSC showing stability, averaging 1.17%. Additionally, one of the basic requirements in agricultural sensing systems is that SHS yield readings are able to capture dynamic movements in water levels in soil based on infiltration rate changes [18], averaging 16.23% across the temporal range, indicating sensor robustness in capturing moisture fluctuations in soil while also maintaining a moving average record to understand irrigation management practices and levels as a whole. In addition to soil moisture information, the Sensor-in-Field probes depict the capability to effectively report soil physical information as SVD, whose values were constant across the 28-day measurement period, averaging 1.32 g cm^−3^.

Coefficient of variation (CV%) for Sensor-in-Field probes data was well within acceptable limits (<20%) for all parameters based on CLSI (Lab standards regulation) [32]. This confirms the stability over time and their multi-functionality, which enables Sensor-in-Field probes to reliably assess soil health properties. Further significance of these capabilities includes the potential to provide real-time insights for actionable agricultural practices and soil management, thereby facilitating improved land management.

The field validation demonstrates the need for comprehensive, continuous, and accurate monitoring of critical soil parameters influencing soil health. The study findings clearly show the temporal variability of soil parameters, highlighting the need for SODS to be implemented in farms for high resolution data collection. Precision agriculture could leverage the high-resolution data from SODS-based Sensor-in-Field probes to improve variable rate nutrient application, site-specific irrigation, and crop health monitoring, and predictive models can be used to enhance efficiency, sustainability, and yield.

### 3.1. Validation by Parameter

#### 3.1.1. Soil Organic Matter (SOM)

SOM is a critical indicator of soil health and fertility. The Sensor-in-Field probes [23] measured an average SOM content of 1.9%, closely matching the reference laboratory value of 1.98%. With a coefficient of variation (CV%) of 2.8%, the Sensor-in-Field probes demonstrated excellent precision in their readings. Bland–Altman analysis showed an average of 4% difference between the sensor’s data and lab analysis, which is well below the 20% accuracy limit. Reliable SOM measurements are vital for understanding soil structure, nutrient cycling, and overall soil fertility. The study’s results validate the use of Sensor-in-Field probes for accurately monitoring SOM and supporting sustainable soil management practices.

#### 3.1.2. Total Soil Carbon (TSC)

The accuracy of Sensor-in-Field probes in measuring total soil carbon (TSC), which is the total of Soil Organic Carbon and Soil Inorganic Carbon, was validated by comparing their readings to reference laboratory values. The probes recorded an average TSC of 1.17%, while the reference method showed 1.19%. With a coefficient of variation (CV%) of 1.9%, the Sensor-in-Field probes demonstrated low variability and high accuracy in their measurements. Similarly, Bland–Altman analysis shows an average of 4% difference between the sensor’s data and lab analysis, which is well below the 20% accuracy limit. Accurate TSC data are crucial for assessing soil health and fertility, supporting effective carbon sequestration practices. The study’s findings affirm that Sensor-in-Field probes are reliable, one-of-a-kind tools for monitoring total soil carbon, providing valuable insights for sustainable soil management strategies.

#### 3.1.3. Soil Volumetric Density (SVD)

Soil volumetric density (SVD) measurements obtained using the Sensor-in-Field probes were compared to those from laboratory analysis to validate their accuracy. The Sensor-in-Field probes recorded an average SVD of 1.317 g/cm^3^, very close to the reference value of 1.32 g/cm^3^. The CV% for the Sensor-in-Field probe readings was 1.5%, indicating very low variability and high reliability. Bland–Altman analysis shows an average of 1% difference between the sensor’s data and the lab analysis, which is well below the 20% accuracy limit. Accurate SVD data are crucial for understanding soil compaction, which affects water holding capacity, root penetration, and nutrient diffusion. The study confirms that Sensor-in-Field probes are highly effective for measuring soil density, providing essential data for soil health management.

#### 3.1.4. Carbonaceous Soil Minerals (CSMs)

The validation study assessed the accuracy of Sensor-in-Field probes in measuring carbonaceous soil minerals (CSMs) [24], a crucial parameter reflecting total soil inorganic carbon content (carbonates and bicarbonates), which is essential for microbial activity and overall soil health and is often left out of environmental models due to difficulties with measurement and a lack of understanding of this vital soil carbon pool. The Sensor-in-Field probes recorded average CSMs of 0.049%, while the reference laboratory method reported an average of 0.044%, as shown in Figure 4a. *T*-test analysis between the lab and sensor data shows a *p*-value > 0.005, indicating a non-significant difference. The consistency between these two methods demonstrates the probes’ reliability in capturing soil inorganic carbon levels. Additionally, the Sensor-in-Field probes exhibited a coefficient of variation (CV%) of 3.1%, indicating consistent precision in their measurements. Accurate CSM data are vital for assessing soil resilience and productivity, as carbon content plays a significant role in nutrient availability and soil microbial dynamics. The study’s findings confirm that Sensor-in-Field probes can effectively monitor CSM, providing essential insights for sustainable soil management and agricultural practices.

#### 3.1.5. Ammonium (NH_4_)

The validation of Sensor-in-Field probes for ammonium (NH_4_) measurements [20] involved comparing probe readings with those obtained from laboratory analysis. The Sensor-in-Field probes recorded an average ammonium concentration of 2.78 ppm, while the reference method reported 2.46 ppm, as shown in Figure 4b. *T*-test analysis between the lab and sensor data shows a *p*-value > 0.005, indicating a non-significant difference. This high degree of correlation confirms the probes’ effectiveness in measuring NH_4_ levels. The probes demonstrated a CV% of 4.2%. Accurate ammonium data are essential for managing nitrogen uptake and balancing soil nutrients, ensuring optimal crop development.

#### 3.1.6. Nitrate (NO_3_)

The study meticulously evaluated the Sensor-in-Field probes’ capability to measure nitrate (NO_3_) levels in soil [19]. On average, the Sensor-in-Field probes recorded a nitrate concentration of 4.40 ppm, compared to the reference laboratory value of 3.88 ppm, as shown in Figure 4c. *T*-test analysis between the lab and sensor data shows a *p*-value > 0.005, indicating a non-significant difference. This close alignment underscores the probes’ precision in detecting nitrate levels, which are crucial for understanding nitrogen availability in the soil. The coefficient of variation (CV%) for the Sensor-in-Field probe readings was 3.6%, highlighting their low variability and high accuracy. Given that nitrate is a key nutrient for plant growth, the ability of the Sensor-in-Field probes to reliably monitor NO_3_ levels is vital for optimizing nitrogen management in agricultural practices.

#### 3.1.7. Soil Hydration State (SHS)

The study also validated the Sensor-in-Field probes’ performance in measuring soil hydration state (SHS), which is critical for effective irrigation management. The Sensor-in-Field probes recorded an average SHS of 16.23%, compared to the laboratory’s average of 15.42%. The coefficient of variation (CV%) for the probe readings was 2.2%, as shown in Figure 4d. *T*-test analysis between the lab and sensor data shows a *p*-value > 0.005, indicating a non-significant difference. Accurate SHS measurements guide irrigation practices, ensuring optimal soil moisture for root growth and nutrient uptake. The consistent and precise readings provided by the Sensor-in-Field probes against the standard methodology, while also contributing to capturing the dynamic movement of water into the soil structure [18], confirm their utility in real-time soil moisture monitoring, essential for maintaining healthy crop growth.

### 3.2. Soil Contextual Modeling

SOC and SIC (CSM) sensor measurement data were compared to a predictive model. A modeling framework was developed using an ensemble of machine learning methods to predict average and attainable soil carbon levels, and average water holding capacity for the continental United States [33,34]. These models can predict SOC and SIC stocks in a 1 m soil profile to a spatial resolution of 250 m^2^. Average SOC and SIC projections represent the expected contemporary baseline at a given area, contextualized based on the location, soil type, climatic conditions, and land use practices, whereas model projections for attainable SOC and SIC depict the likely maximum levels achievable at a given location. For the area where the sensors were placed, the contemporary baseline models predicted SOC and SIC to be 114.08 t ha^−1^ and 33.85 t ha^−1^, respectively. The attainable SOC stock in the 1 m soil profile was projected to be 182.56 t ha^−1^. The average water holding capacity was projected to be at 13.843 mm.100 mm^−1^ of soil. Based on Sensor-in-Field TSC and CSM measurements at a depth of 20 cm, the stocks of SOC and SIC in the 1 m soil profile were estimated to be 149.16 t ha^−1^ and 6.453 t ha^−1^, respectively. The sensor measurement depth probably explains some differences between the model and the SIC sensor measurement. Generally, SOC tends to decrease with soil depth [35], whereas SIC likely increases at very low levels at the sensor measurement depth [36]. Moreover, the baseline model projections were for the year 2020. The estimated SOC stocks from the Sensor-in-Field measurements are within the range expected by the modeling framework.

### 3.3. Sensor Accuracy and Reliability

Table 2 details the statistical analysis of the measured soil health metrics, where multiple conclusions can be drawn. First, the coefficient of variance observed in the carbon moieties is much lower (<7%) when compared to that of the nutrients (17–20%). This is expected, since a 1% change in either organic or inorganic carbon could take decades, while nutrients vary more rapidly, mostly due to fertilization. Although this was an unmanaged field where no fertilizer was added, the fluctuations due to the wheat plant’s nutrient uptake from the soil were captured throughout the 28 days, showing both nitrate and ammonium varying around the moving average. Second, all parameters showed different patterns, although the moisture content alternated between 13% and 20%. This indicates that the sensors are immune to moisture content as long as the electrochemical cell is complete. During the calibration studies, the sensor’s accuracy was unaffected in the moisture range from ~10% to 80%, depending on soil texture, where the higher clay content reduces the fluctuations compared to sandy soil.

All sensors had an error rate of less than 20% when compared with the certified soil analysis laboratory’s discrete sampling points T1, T2, and T3 marked on the time series plots in Figure 4 and Figure 5. Table 3 summarizes the sensors’ measured concentrations and the concentrations measured at the soil analysis laboratory, as well as the error rate between the two methods for all parameters. The carbons had a lower error rate than the nutrients due to the volatility of the nitrogen moieties compared to the more resilient carbon moieties.

### 3.4. Technology Limitations

Due to the vastness of soil matrices, we quickly learned that different soil textures needed to be treated like different samples if we were to compare Earth to Humans. Sandy soil can be viewed as exhaled breath—barren of everything and has low contents of carbon, nutrients, and organic matter. Clay soil, on the other hand, is similar to blood—has a high content of organic matter and adequate levels of other minerals and nutrients, making it a more complex matrix than sandy soil. This was evident in the data from the calibration models for different soil textures that were published in the lab’s previous papers [19,20,23,24,37,38,39]. The other soil textures exhibited slightly different calibration models from the loam soil presented in Figure 1, which was used to calculate the concentrations measured in this study.

### 3.5. Future Work

The field used in this study was an unmanaged field growing winter wheat. The data collected only showcase the robustness and accuracy of Sensors-in-Field probes. Future field studies will include a negative control field. Part of the field will be actively managed, and the other side will have no external inputs or growing crops. This experiment will serve two purposes. First, the measurements from the unmanaged control field will provide fluctuations solely due to environmental or hardware factors, whereas the actively managed field will capture the effects of current farming techniques and methodologies, as well as microbial activity in the presence of plant roots on top of the environmental and hardware-induced fluctuations. Second, as both fields are in the same geographical location and data are collected simultaneously, the measurements from the control field can be treated as the background noise for the actively managed field. By filtering out the background noise, farmers would be able to judge the true impact of their farming techniques and methodologies, finally helping to answer the question “How do modern agriculture techniques affect soil health?” using accurate, real-time data.

## 4. Conclusions

The validation study confirmed the high reliability and accuracy of Sensor-in-Field probes in measuring soil parameters, aligning closely with reference laboratory methods. The probes demonstrated low coefficients of variation (CV%) across all parameters, ranging from 1.5% to 4.2%, indicating excellent precision. Key nutrient measurements included nitrate (4.40 ppm vs. 3.88 ppm), ammonium (2.76 ppm vs. 2.46 ppm), inorganic carbon (0.049% vs. 0.044%), and soil organic matter (1.9% vs. 1.98%). These findings validate the robustness of Sensor-in-Field probes for real-time soil monitoring, which is essential for precision agriculture. Regular use of these probes can enhance nutrient management, irrigation practices (by means of utilizing SHS and SVD sensor data), and crop productivity, supporting data-driven agricultural decisions and sustainability. The authors acknowledge that the sensors’ calibration models need to be refined with more data from different fields, especially across continents. This will further enhance the robustness of the probe to account for other unstudied soil factors that can affect the sensors’ performance.

## Figures and Tables

**Table 1:** Summary of the plot information, weather conditions, and mean soil health metrics measured by the Sensor-in-Field probes.

Site Coordinator: Donald Danforth Plant Science Center			
Location	St Charles, MO 63301, USA		
GPS Coordinates	38.846968, −90.462104		
Study Period	7 November 2024 to 5 December 2024		
Days	28		
# of Cycles	84		
Temperature range (°C)	−4 to 28		
RH range (%)	18–90		
Soil type	Silt Loam		
Sensor’s Depth	15 cm		
Parameter	Average	Std. dev.	CV (%)
CSMs (SIC) (%)	0.0	0.0027	5.5
SOM (%)	1.92	0.021	1.1
TSC (SOC + SIC) (%)	1.18	0.081	6.9
NO_3_ (ppm)	4.44	0.89	20
NH_4_ (ppm)	2.78	0.48	17
SVD (g/cm^3^)	1.32	0.023	1.7
SHS (%)	15.88	2.1	13

**Table 2:** Summary of the statistical analysis for all soil health metrics measured by the Sensor-in-Field probes.

	CSM (%)	SOM (%)	TSC (%)	Nitrate (ppm)	Ammonium (ppm)	SVD (g/cm^3^)	SHS (%)
Minimum	0.045	1.9	1.0	2.7	1.8	1.3	13
Maximum	0.054	1.9	1.4	6.0	3.5	1.4	20
Range	0.0090	0.090	0.36	3.3	1.6	0.070	6.7
Mean	0.049	1.9	1.2	4.4	2.8	1.3	16
Std. Deviation	0.0027	0.021	0.081	0.89	0.48	0.023	2.1
Std. Error of Mean	0.00051	0.0039	0.015	0.17	0.090	0.0043	0.40
Coefficient of variation	5.5%	1.1%	6.9%	20%	17%	1.7%	13%

**Table 3:** Summary of the sensors’ measured concentrations and the concentrations measured at the soil analysis laboratory, as well as the error rate between the two methods.

Parameter	Sensors-in-Field	Soil Lab.	Error Rate (%) *
CSM (SIC) (%)	0.049	0.044	11.36
SOM (%)	1.92	1.98	3.03
TSC (SOC + SIC) (%)	1.18	1.19	0.84
NO_3_ (ppm)	4.44	3.88	14.43
NH_4_ (ppm)	2.78	2.46	13.01
SVD (g/cm^3^)	1.32	1.32	0.21
SHS (%)	15.88	15.42	2.98

* Calculated using (|Sensors − Lab|/Lab) × 100, taking the lab report as the true reference.

## Data Availability

Data can be shared only with the sponsor’s permission.
